# Evaluation of marginal sealing quality of restorations with low shrinkage composite resins

**DOI:** 10.4317/jced.57402

**Published:** 2020-12-01

**Authors:** Bruno-Mendonça-Lucena de Veras, Renata-Pedrosa Guimarães, Luiz-Carlos Alves, Rafael-José-Ribeiro Padilha, Luana-Osório Fernandes, Carlos-Menezes Aguiar

**Affiliations:** 1 PhD student - UFPE - Federal University of Pernambuco, Department of Prosthetic Dentistry and Maxillofacial Surgery, 1235 Professor Moraes Rego Ave, Recife, PE, 50670901, Brazil; 2 PhD, Associate teacher -UFPE - Federal University of Pernambuco, Department of Prosthetic Dentistry and Maxillofacial Surgery, 1235 Professor Moraes Rego Ave, Recife, PE, 50670901, Brazil; 3 PhD, Associate teacher - UFPE - Federal University of Pernambuco, Department of Electronic Microscopy of the Laboratory of Immunopathology keizo Asami – Lika, 1235 Professor Moraes Rego Ave, Recife, PE, 50670901, Brazil; 4 MSc, Associate microscopy technician) - UFPE - Federal University of Pernambuco, Department of Electronic Microscopy of the Laboratory of Immunopathology keizo Asami – Lika, 1235 Professor Moraes Rego Ave, Recife, PE, 50670901, Brazil; 5 PhD student - UFPE - Federal University of Pernambuco, Department of Prosthetic Dentistry and Maxillofacial Surgery, 1235 Professor Moraes Rego Ave, -Recife, PE, 50670901, Brazil; 6 PhD, Associate teacher - UFPE - Federal University of Pernambuco, Department of Prosthetic Dentistry and Maxillofacial Surgery, 1235 Professor Moraes Rego Ave, Recife, PE, 50670901, Brazil

## Abstract

**Background:**

This study compared the quality of marginal sealing in the gingival wall of class II preparations of two low-shirinkage resins of the bulk fill type with a conventional resin isolated or associated with a glass ionomer cement (GIC).

**Material and Methods:**

40 human molars were divided into 4 groups and 80 occlusal-mesial and occlusal-distal restorations were performed with the following materials: SureFil SDR flow, Filtek Bulk Fill Posterior, Z250 resins and Riva Light Cure GIC. 40 restorations were evaluated in Scanning Electron Microscopy (SEM) with Elemental Microanalysis Spectrometry (EDS) initially and the remainder after a period of 6 months of aging in a 37 ± 5°C oven. An average of the silver penetration at each restoration was obtained in the two evaluations and the results were statistically analyzed in a descriptive and inferential way, through the paired t-Student and one-way ANOVA F-test.

**Results:**

There were no significant statistical differences between the materials with respect to silver nanoinfiltration, except for the Bulk Fill Posterior/3M ESPE resin compared to the GIC and conventional resin in the final evaluation.

**Conclusions:**

The low shrinkage resins showed a similar behavior in relation to the marginal sealing quality observed in the GIC or composite resin with the incremental technique, also presenting the advantage of simplicity in the technique of confection of the restorations and reduction of the time of work.

** Key words:**Resin composites, Bulk fill, dental restorations, marginal quality, adhesion.

## Introduction

Dental composite resins are the materials of choice for restorations of teeth in times that aesthetic requirements or values influence the choice of the professional and the patients seeking dental treatments (1).

Despite the evolution of adhesive techniques and the properties of these restorative materials, which allow their safe use in posterior teeth (2-5), some problems, such as the polymerization contraction, persist. This, eventually, leads to cracks and consequent marginal infiltration, which is associated with postoperative sensitivity, marginal discoloration and secondary caries, being the main causes of replacement of these restorations (6,7).

The magnitude of the contraction depends on the resin matrix formulation, the viscoelasticity of the dental composite and the insertion technique used in the restorative treatment (4,5). Factors that are directly related to the integrity and quality of the marginal sealing (6).

In order to overcome the problems related to the contraction of these materials and their consequent marginal infiltration, several steps are proposed such as the control of the cavity conFiguration factor (C-Factor), the incremental insertion technique, optimization of the polymerization method and the addition of intermediate layers of lower modulus of elasticity materials (8,9).

Although the incremental technique is preferred for use with methacrylate based resins, some disadvantages are pointed out, especially when considering its application to proximal cavities, such as the incorporation of voids into the restorative mass, contamination and failure of adhesion between layers, difficulties in insertion of increments in areas of difficult access, and extensive treatment time (9,10,6).

The association of glass ionomer cement (GIC) with composite resin has been suggested as the best option for restorations with composite resins in proximal cavities of posterior teeth. It may be justified for it’s ability to chemically react with the calcium of the tooth structure, providing a more effective and long lasting seal, besides of presenting modulus of elasticity and coefficient of thermal expansion similar to dentin (11).

A new type of resins called bulk fill was introduced in the last few years. Modifications in the chemical structure of the matrix, with the use of lower viscosity monomers and incorporation of photoactive groups called “polymerization modulators”, allow, according to manufacturers, these materials to be used in increments from 4 mm to 5 mm in class I and II cavities. This promotes a more adequate and effective marginal sealing given the better wetting of the surface, degree of polymer conversion, lower stress and polymerization contraction (12-14,1). Moreover, the elimination of the multiple steps in the incremental technique associated or not to the GIC contributes to a shorter clinical time, greater simplicity and less probability of errors in the restorative process.

Faced with the search for restorative more sTable materials in the oral cavity and the limited amount of studies that evaluate the success of the low contraction stress resins, it becomes evident the need to unveil the real benefits that this new modality of dental composite may provide to the patient and to the clinical practice of dentists.

The present study aimed to compare, *in vitro*, through nanoinfiltration, the quality of marginal sealing in the gingival wall of class II cavities, with absence of enamel, of two low fill resins of the bulk fill type with a conventional composite resin isolated or associated with glass ionomer cement (GIC) - open sandwich technique.

## Material and Methods

The Research Ethics Committee of the Federal University of Pernambuco approved this study (Approval number: 1.619.548).

It was carried out an *in vitro* experimental study at the Federal University of Pernambuco (UFPE). A total of 40 healthy third molars were selected from the teeth bank of the UFPE, ranging from 18 to 40 years old, free of fractures, cracks, macroscopic defects and preferably selected so that the occlusal and cervical height of the proximal faces were close to or exactly 6mm.

The collected specimens were stored in containers with 0.5% Chloramine solution at room temperature for a period of 7 days and then, in distilled water with weekly changes until the time of their use, which did not exceed the period of six months.

In each specimen, two proximal cavities were prepared involving the mesial-occlusal and distal-occlusal surfaces, totaling 80 cavities. An array made of polyester strip and 4x6mm opening was used to standardize the wells. This matrix was used as reference for demarcating, with the help of a hydrographic pen, the external contour of the cavities on the faces of the specimens.

The cavities were standardized: 4 mm wide in the buccal-lingual direction, 6 mm high proximal box and 2 mm axial depth. In all the preparations, the cervical term was located beyond the cementoenamel junction in dentin and cement, and the measurements were verified with the use of a millimeter probe.

In the cavities where the cervical-occlusal distance exceeded 6mm, the occlusal surface of the specimens was worn until the distance remained exactly 6mm. Cavity preparations were performed by the same operator, using a cylindrical diamond bur (#4137 – KG SORENSEN, Cotia, Brazil), in high speed, under constant cooling with water/air spray. Every ten usages, the diamond bur was replaced.

After the cavity preparation procedures, to allow a restorative process close to the conditions found in the oral cavity, the specimens were fixed in a simulator and secured with utility wax. The Tofflemire matrix, with wooden wedge, was also adapted for all specimens to be restored.

Prophylaxis was performed with pumice stone (SSWhite, Rio de Janeiro, Brazil) and water, with the aid of a robinson brush (Microdont, São Paulo, Brazil). After, the cavities were washed and dried with slightly moistened cotton pellets. The dental surface conditioning was carried out with 37% phosphoric acid, respecting the time of 30 seconds in enamel and 15 seconds in dentin.

The 40 specimens were previously randomly distributed in 4 groups and restored with the materials and techniques described in  Table 1, according to the manufacturers’ recommendations, totaling 80 restorations, with 20 restorations per group.  Table 2 shows information regarding the composition of restorative materials used.

**Table 1:**
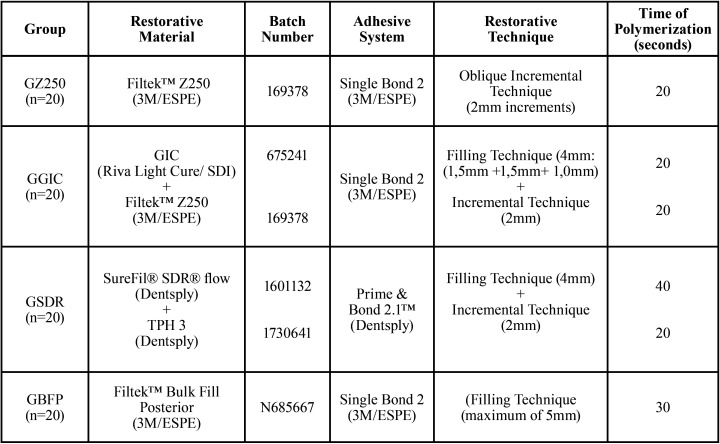
Distribution of restorative systems, adhesives and restorative technique by group.

**Table 2:**
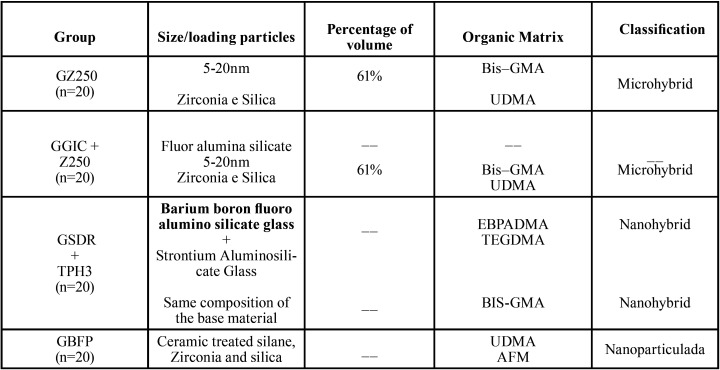
Composition of the restorative materials.

The photopolymerization was carried out using a high-intensity LED device (Radii-cal / sdi, 1200 mW/cm2 power and 460 nm λ), for a period of time recommended by the manufacturer ( Table 1) for each material at a minimum possible distance from the polymerized restorative material, initially by the occlusal and later by buccal and lingual surfaces. After all restorations and removal of the matrix system were performed, the coarse excesses were removed with scalpel blades and composite resin strips (3M ESPE, St Paul, USA).

Afterwards, for the hygroscopic expansion of the composite resin to occur, the teeth were stored in distilled water with a temperature ranging from 35 ± 7°C in a greenhouse for 24 hours. Then, the restorations were submitted to the final finishing and polishing procedures with Sof-lex Pop-on discs (3M ESPE, St Paul, USA) in descending order of abrasiveness, until no excess was observed, and subsequently diamond felt discs (FGM, Joinville, Brasil) associated with the polishing paste Diamond R (FGM, Joinville, Brasil), until a smooth and homogeneous proximal surface was obtained. The disks were replaced every five finished and polished restorations.

Thermocycling was performed on all specimens with 250 cycles in water between 5 ± 5°C and 55 ± 5°C temperature. The exposure in each bath was 20 seconds and the transfer time between the baths was 3 to 5 seconds.

Five randomly selected specimens from each experimental group (10 restorations) were prepared for the initial analysis of nanoinfiltration in scanning electron microscopy. For this, all surfaces of the specimens were waterproofed with two layers of nail polish (Risqué, Goiânia, Brazil) on all their faces except a distance of 1.0 mm of the mesial and distal cervical wall of each specimen observed.

The specimens were then immersed in a marker solution containing 50 wt. % ammoniacal silver nitrate (pH 9.5) for 24 hours at room temperature. After this period, they were washed in distilled water, and immersed in a developer solution for 8 hours in fluorescent light to reduce the diaminoprotein ions to metallic silver grains. At the end of 8 hours, the specimens were washed in running water.

Subsequently, the specimens were sectioned, mesio-distally in the sagittal plane, with the aid of a double-sided diamond disk coupled to a low-speed precision cutter (Buehler IsoMet® Low Speed Saw, Binghamton, New York, USA). They were polished in a polisher in order to remove scratches and irregularities. It was also used silicon carbide sandpaper in decreasing order of abrasiveness (# 600, # 1200) for a period of 20 seconds and washed abundantly with each exchange of sandpapers.

The accurate visual analysis of each face obtained after the sagittal cut of the teeth, as well as the presence of fractures or maladaptation of the restorative material, directed the choice by the face that presented the best conditions for analysis.

For the analysis in Scanning Electron Microscope (SEM), the specimens were submitted to the following protocol:

- Acid conditioning of the tooth-resin interface with 37% phosphoric acid for 5 seconds, followed by washing with water for 10 seconds;

- Immersion in 2.5% sodium hypochlorite solution for 2 minutes;

- Ultrasonic bath for 20 minutes to remove any residues on the cut surface;

- Dehydration in ascending degrees of acetone: 25%, 50%, 75%, 95% (20 minutes at each concentration) and 100% (60 minutes).

The specimens were then stored in a greenhouse for 72 hours at a temperature of 37 ± 5°C, fixed in stubs with double-face carbon tape, and silver enamel (Bal- Tec - Balzers, Liechtenstein) was applied. (Quorum 150T ES, Laughton, USA), with a pressure of 0.05 mbar, a current of 20 mA, a working distance of 50 mm, a coverage time of 60 seconds and a mean thickness of 20 nm exposure.

**Figure 1:**
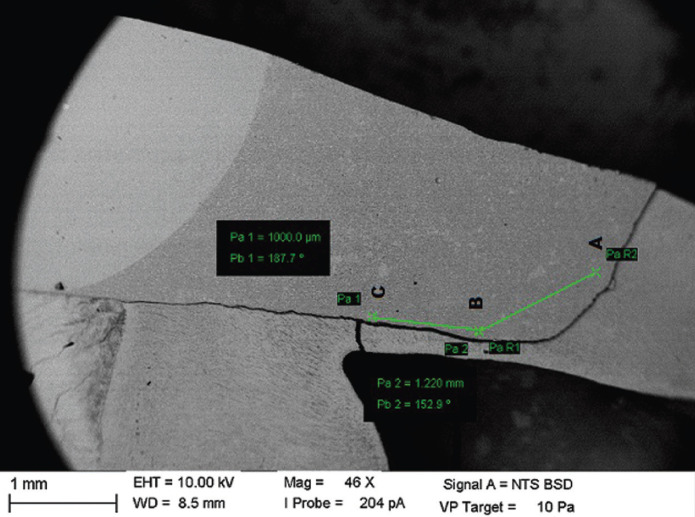
Photomicrograph of the marking points to be magnified. A: 1mm of distance of the beginning of the restoration. B: 2mm of distance. C: 3mm of distance.

**Figure 2:**
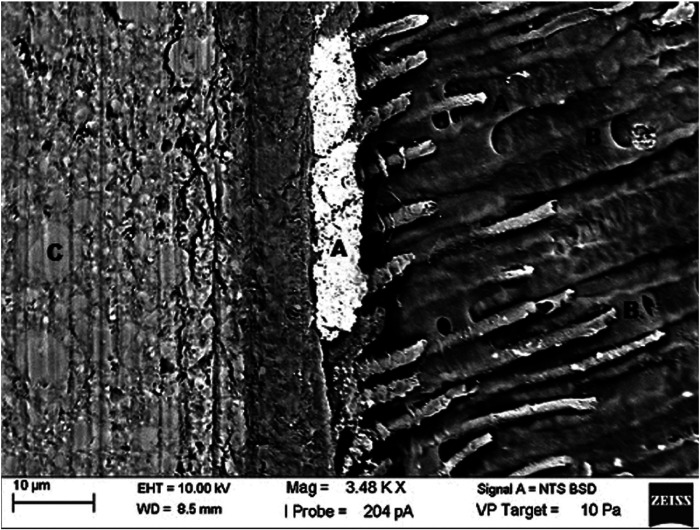
Magnification photomicrography standard for analysis in EDS of the GZ250. A: Penetration of silver in the hybrid layer, tags and dentinal tubules. B: Dentinal tubules. C: Z250 3M/ESPE dental composite resin.

The SEM analysis (Carl Zeiss - EVO MA15, Oxford Instruments) was performed on dentin, cervical and axial walls of the mesial and distal surfaces of the 5 restored specimens of each group, totaling 10 restorations initially evaluated per group. The photomicrographs obtained of each restoration were standardized, so that three points at the tooth-restoration interface, distant 1mm, 2mm and 3mm from the beginning of the restorations were marked with an increase of 46x (Fig. 1), enlarged from 2000x to 3500x (Fig. 2) and analyzed for the maximum percentage of silver present in each region, through the EDS (IncaWave Oxford Elemental Microanalysis Spectrometer) coupled to the SEM.

It were obtained averages from the percentages of silver found in the three marked and enlarged points in order to establish the degree of silver penetration in each restoration.

For the evaluation after aging, the five other specimens from each group (n = 10) not initially evaluated were analyzed after a period of six months of storage in a laboratory oven at 37 ± 5°C, following the same protocol of the initial evaluation.

The results, obtained from the initial and post-aging evaluations, were statistically analyzed in a descriptive and inferential manner.

The descriptive analysis was based on mean, standard deviation and median. The inferential analysis was performed using the paired Student t test (for the comparison between the evaluations in each group) and F (ANOVA) with one factor (for comparison between groups). In the case of significant difference by the F test (ANOVA) the Tukey multiple comparisons (among pairs) were obtained.

The verification of the hypothesis of normality of the data was performed by the Shapiro-Wilk test and the equality of variances was through the Levene F test. The margin of error used in the statistical test decisions was set at 5%. The software used to obtain the statistical calculations was SPSS (Statistical Package for the Social Sciences) in version 23.

## Results

The results are shown in  Table 3, which presents the means, standard and median deviations of the percentage of silver impregnated in the restorations according to the material employed and the evaluation periods, as well as the average of the variations between evaluations of the final value (post aging) minus the initial value.

**Table 3:**
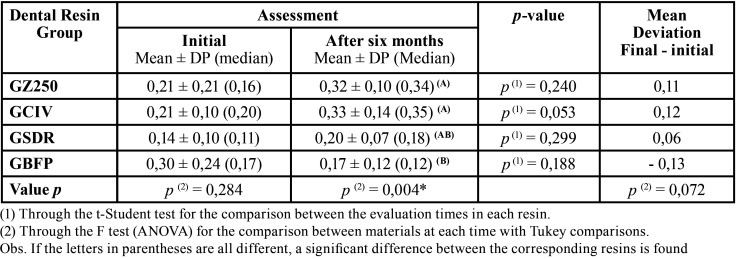
Percentage of silver according to the material used and evaluation of the variation between the periods.

In the initial evaluation, the greatest infiltration occurred for GBFP, while the lowest infiltration averages were observed in GSDR. In the final evaluation, the highest means occurred in GCIV (0.33) and in GZ250 (0.32). The mean variation was - 0.13 (reduction) for GBFP and ranged from 0.06 to 0.12 in the other groups. As can be observed, with the exception of GBFP, whose average had a reduction from the initial value to the end of 0.30 to 0.17, the others or groups showed an increase in the average of silver infiltration in the restorations after the aging period.

Among the resins, significant differences were recorded in each of the evaluations, except for the Filtek Bulk Fill resin (3M ESPE, St Paul, USA) compared to the Filtek Z250 resin (3M ESPE, St Paul, USA) and the Cement Riva Light Cure Glass Ionomer (SDI, Victoria, Australia) during the final evaluation, which showed a significant difference (*p* <0.05) verified by the Tukey test.

## Discussion

Adhesion to dentin is totally dependent on variables inherent to its surface, such as the orientation of tubules, water content, presence or absence of smear layer and permeability. In addition, a correct technique of applying the adhesive systems, avoiding excessive drying and ensuring the infiltration of the monomers in the network of collagen fibers exposed after the acid conditioning, prevent the degradation of the hybrid layer through hydrolysis and promote a more durable and effective adhesion.

Current dentin adhesive systems rely on a complex combination of micromechanical retention by penetration into the partially opened dentin tubules, formation of a hybrid layer and chemical interactions involving primary and secondary bonds. Thus, the limiting factor in the current adhesive restorations seems to be centered in the tensions generated during the polymerization contraction of the composite resins (7).

With the objective of promoting the relief of these tensions, the reduction of the contraction of polymerization in proximal cavities and guided by the adhesive properties and coefficient of thermal expansion similar to the tooth, some authors obtained better results with the GIC used as a basis in the cervical wall of these restorations , which characterizes the so-called “sandwich technique” (16,17).

In this study, GGIC using the same technique presented similar performance in relation to the composite resins (GZ250, GSDR and GBFP) as base material during the evaluations, which corroborates with the studies of Haller and Trojanski (18) and Güngör *et al.* (19), who showed no improvement with the use of a base in GIC in comparison with adhesive systems used with conventional resins.

The consensus in direct comparisons of studies using glass ionomer cements is difficult to obtain, since there is a wide range of materials available with different formulations and characteristics (20). In addition, in this study, the use of healthy teeth and younger patients may have reduced the possibility of better sealing of these materials in relation to the adhesive systems used in association with composite resins due to the lower probability of the presence of dentin sclerosis induced by stimuli to this substrate which is more common in senile or affected teeth by carious lesions, where the performance of the material would be optimized.

The initial evaluation showed a similar performance between the G250, GSDR and GBFP groups, with no statistically significant difference between them. These results were also found by Campos *et al.* (6) and Roggendorf *et al.* (21), that studied the marginal sealing quality in conventional resins with incremental technique and bulk fill resins in class II cavities and did not find differences when the cavity configuration factor was altered.

The marginal sealing quality and the absence of gaps in composite resin restorations is highly dependent on the C-factor, which is represented by the relationship between the adhered surface areas and the areas not adhered by the resin in the cavity. The increase of this ratio, expected in single fill resins, causes increase of residual stress and higher degrees of polymerization contraction, whereas, it is expected to reduce this factor in incremental techniques (15). In this study, the increase of the cavity configuration factor present in GSDR and GBFP did not contribute to the increase of silver nanoinfiltration in the initial evaluation, which may be related to the new monomers (EBPADMA E AFM, respectively) and polymerization modulators inserted by the manufacturers In the resin matrix formulation. This makes the use of these materials more advantageous, considering the simplicity in the technique of performing the restorations and the shortest working time required.

In the post-aging evaluation, only the GBFP group in relation to GZ250 and GCIV showed a smaller and statistically significant penetration of silver (*p* <0.05). In addition, it was the only group that presented a reduction of the concentration mean in relation to Initial evaluation, despite the absence of statistical significance.

Although in the adhesion studies the specimens tend to show better results in marginal sealing in initial evaluations and to diminish their efficiency in post-aging evaluations, due to the degradation of the union attributed mainly to the hydrolysis of the resinous components (15), a change in this behavior can occur front to the storage in the presence of water, which can cause a reduction of nanoinfiltration by hygroscopic expansion of the composite (22). Considering that during the first GBFP evaluation showed the highest averages of silver infiltration, it can be assumed that the material may have been influenced Hygroscopic expansion in the post-aging evaluation.

**Figure 3:**
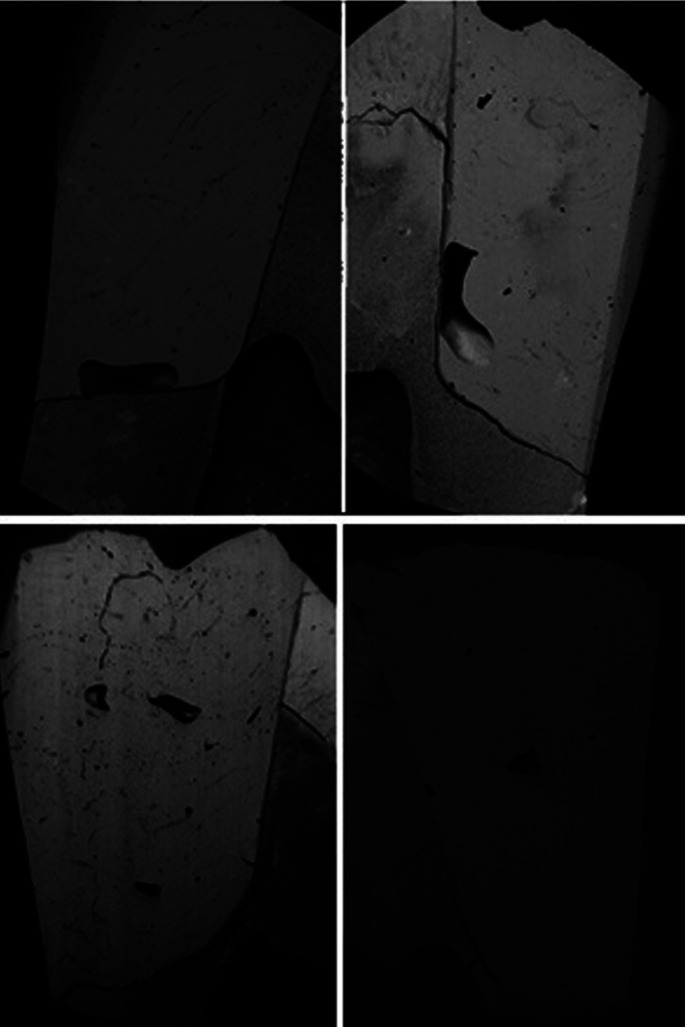
Photomicrography showing presence of bubbles in restorations of the group GBFP.

Another important finding in this study in relation to the GBFP group was the presence of blisters, even though the manufacturer’s recommendations regarding the insertion technique, common in some specimens of the initial and final evaluation (Fig. 3), were respected. This finding leaves gaps regarding the longevity of these *in vivo* restorations, indicating the need for further studies and randomized clinical trials.

It is known that in addition to factors related to the operator, the success of adhesive restorations is also dependent on the physicochemical characteristics of the materials used and the restorative technique used, and a higher contraction tension and internal porosity in resin restorations is expected. The volume inserted into a single increment (7,23).

We suggest the use of a composite resin of lower viscosity and low contraction prior to the use of medium viscosity Bulk Fill resins (GBFP), since, as observed in GSDR, material adaptation can be optimized by reducing the incidence of bubbles, Which indicate cohesive failures and may be responsible for the increase in marginal infiltration and consequent incidence of postoperative sensitivity, in addition to loss of restoration in the short term (15,23).

Although there were no statistically significant intragroup differences or between the groups considering the two evaluations, a better result was observed for the GSDR group, which presented a lower average variation of the silver penetration in relation to the other groups, showing a better Stability of the hybrid layer after the aging period.

Such a finding may be the result of a lower polymerization contraction of the restorative material, which has a polymerization modulator which according to the manufacturer would be responsible for the reduction of stresses through prolongation of the pre-gel phase. In addition, its self-leveling ability and the use of the acetone-based bonding agent in the restorative process causes a decrease in the presence of voids not infiltrated by the monomers and promotes the formation of a more stable hybrid layer (15).

The studies of the physical-mechanical properties of interfaces and nanoinfiltration have shown to be important and complementary tools to estimate the longevity of restorative materials in the oral cavity (21,24-26). The occurrence of nanoinfiltration alone does not predict the occurrence of early and early adhesives failure of the restorative material or bacterial infiltration because the pores that allow its occurrence are not large enough for bacterial penetration but allow the passage of its acid metabolism. Which in the long term causes adhesive failures, compromising the marginal sealing quality of the restorations (15).

**Figure 4:**
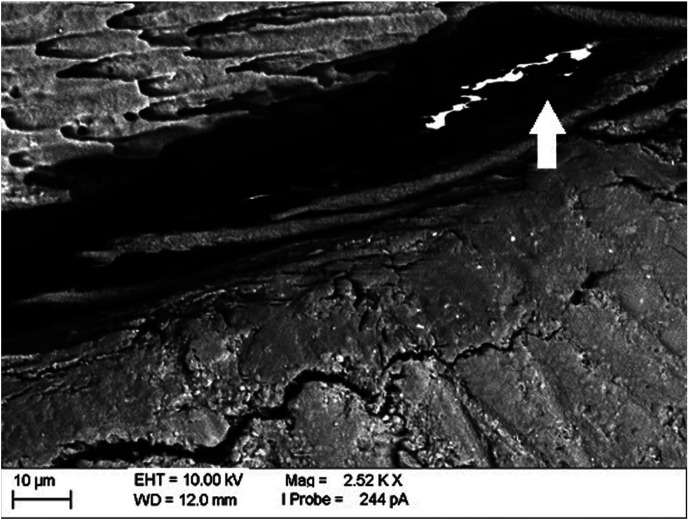
Photomicrograph of the marking points to be magnified. A: 1mm of distance of the beginning of the restoration. B: 2mm of distance. C: 3mm of distance.

The main advantage of studying the performance of restorative materials in marginal sealing by the technique of nanoinfiltration is the possibility of using EDS in the elemental microanalysis of the marker used, usually AgNO3 (silver nitrate), avoiding erroneous interpretations of the presence of the ion Provided by the SEM images, as can be seen in Figure 4.

The findings of this study suggest that clinical research is necessary to verify the real benefits that these new materials will provide in the clinical day-day of the dental surgeon and his patients, since, in showing results *in vivo*, they provide support for making a more accurate decision therapy.

## Conclusions

The low contraction resins of the Bulk Fill type showed similar behavior in relation to the quality of the marginal sealing observed by the Glass Ionomer Cement or the conventional composite resin with incremental technique, also presenting the advantage of simplicity in the technique of confection of the restorations and reduction of the time of work.
